# Editorial: Interdisciplinary innovations in CAR T-cell therapy for autoimmune and cancer treatment

**DOI:** 10.3389/fimmu.2026.1780262

**Published:** 2026-01-16

**Authors:** Apostolos Zaravinos, Derya Unutmaz, Marko Radic

**Affiliations:** 1 Department of Life Sciences, School of Sciences, European University Cyprus, Nicosia, Cyprus; 2 Cancer Genetics, Genomics and Systems Biology Laboratory, Basic and Translational Cancer Research Center (BTCRC), Nicosia, Cyprus; 3 Jackson Laboratory for Genomic Medicine, Farmington, CT, United States; 4 Department of Microbiology, Immunology and Biochemistry, College of Medicine, University of Tennessee Health Science Center, Memphis, TN, United States

**Keywords:** autoimmune disease, bone cancers, chimeric receptor, Graves disease (GD), inflammatory bowel disease (1bd), innate immune cells, tumor microenvironment

The clinical success of chimeric antigen receptors (CARs) in treating lymphoid malignancies (1) and their emerging efficacy in autoimmune diseases (2), has fundamentally transformed cellular receptor engineering from a research frontier into a therapeutic reality. This progress is catalyzing a broader reappraisal of the approach to diseases driven by aberrant cellular immunity. Yet realizing the full potential of these technologies demands more than incremental advances in engineering; it requires deeper integration across basic science, translational research, and clinical disciplines spanning oncology, rheumatology, nephrology, and neurology. The need for such convergence, along with the obstacles that impede it, has been thoughtfully examined in several recent reviews (3).

This Research Topic was conceived to bridge the disciplinary boundaries that often separate the scientists and clinicians driving this rapidly evolving field. The goal was to advance engineered receptor therapies toward broader applications in both autoimmunity and cancer. The collected articles report on conceptual advances and original experimental findings across the therapeutic landscape of chimeric receptors.

## Foundational advances in autoantigen-directed therapy

Cheever et al., contribute original data on a chimeric autoantibody receptor (CAAR) that presents the extracellular domain of the thyroid-stimulating hormone receptor (TSHR) as molecular bait to attract and eliminate TSHR-reactive B cells. Their demonstration of CAAR-mediated cytotoxicity against anti-TSHR B cells *in vitro* establishes the critical first step toward CAAR T cell therapy for Graves’ disease. The data provide a proof of concept that exemplifies the precision-targeting logic now being extended across autoimmune conditions.

## Expanding the autoimmune therapeutic repertoire

Three reviews map the growing landscape of cellular therapies for immune dysregulation. Zheng et al. survey advances in both CAR-T and CAR-macrophage platforms for autoimmune diseases, comparing their capacity to selectively eliminate autoreactive immune populations while weighing therapeutic advantages against translational hurdles. Kümmel et al. turn to regulatory T cells, examining how antigen-specific CAR-Treg engineering might restore immune tolerance in inflammatory bowel disease; they outline strategies targeting circulating carcinoembryonic antigen, flagellin, and IL-23R, while candidly assessing developmental challenges. Wu et al. provide a broader synthesis of how CAR-T therapy, born in oncology, is now transforming autoimmune treatment, with CD19-targeted approaches inducing rapid, durable remissions in refractory cases. Their review also surveys emerging CAR-NK, CAAR-T, and CAR-Treg modalities alongside management of cytokine release syndrome and immune effector cell-associated neurotoxicity syndrome.

## Confronting the solid tumor challenge

The transition from hematologic to solid malignancies remains the field’s most formidable frontier. Stavrou et al. assess the current state of CAR-T applications in osteosarcoma and rhabdomyosarcoma, acknowledging the substantial obstacles these tumors present while highlighting promising clinical trials on the horizon. Andreou et al. dissect why solid tumors resist CAR-T therapy so effectively, focusing on how the tumor microenvironment impedes trafficking, infiltration, and antigen targeting. They evaluate strategies to remodel stromal and vascular barriers, enhance CAR-T precision and persistence, and mitigate off-tumor toxicity, offering a roadmap toward more effective, personalized solid-tumor immunotherapies.

## Broadening the cellular toolkit

Finally, Kuo et al. extend the conversation beyond conventional lymphocyte engineering to innate lymphoid cells (ILCs). Their review details ILC subset diversity, tissue residency, and phenotypic plasticity, positioning these cells as both biomarkers and therapeutic targets. Strategies under exploration, ex vivo expansion, pluripotent stem cell–derived ILCs, CAR-engineered ILCs, cytokine modulation, and checkpoint blockade, suggest new avenues for intervention, though challenges of cellular heterogeneity and manufacturing scalability remain.

## Conclusions

The articles assembled in this Research Topic illuminate both the remarkable progress and the formidable challenges that define cellular receptor engineering today. From CAAR-mediated targeting of autoreactive B cells in Graves’ disease to CAR-Treg strategies for inflammatory bowel disease, from the persistent puzzle of solid tumor microenvironments to the untapped potential of innate lymphoid cells, these contributions collectively map a therapeutic frontier in active expansion.

Several themes emerge across this Research Topic. First, the conceptual architecture pioneered in hematologic malignancies is proving remarkably adaptable, yielding new modalities (CAR-macrophages, CAR-Tregs, CAR-ILCs) tailored to distinct pathophysiologies. Second, autoimmune applications are no longer a speculative extension of oncology successes but a clinical reality generating durable remissions in previously refractory disease. Third, the solid tumor challenge demands not incremental refinement but fundamental innovation in how we navigate immunosuppressive microenvironments.

What unites these diverse efforts is a shared recognition: diseases long considered manageable at best, such as certain cancers, autoimmune conditions, or chronic inflammatory disorders, may now be curable through precise cellular reprogramming. Realizing this possibility will require exactly the kind of cross-disciplinary dialogue this Research Topic seeks to foster. We hope these articles serve not as a summary of current knowledge but as a catalyst, stimulating new collaborations between basic science, translational research, and clinical applications across oncology, rheumatology, nephrology, neurology, and beyond.
